# One-Pot Synthesis of Phenylboronic Acid-Based Microgels for Tunable Gate of Glucose-Responsive Insulin Release at Physiological pH

**DOI:** 10.3390/molecules30153059

**Published:** 2025-07-22

**Authors:** Prashun G. Roy, Jiangtao Zhang, Koushik Bhattacharya, Probal Banerjee, Jing Shen, Shuiqin Zhou

**Affiliations:** 1 Department of Chemistry of the College of Staten Island, and The PhD Program in Chemistry of Graduate Center, The City University of New York, 2800 Victory Boulevard, Staten Island, NY 10314, USA; proy@gradcenter.cuny.edu (P.G.R.); jzhang8@gradcenter.cuny.edu (J.Z.); koushikchem5@gmail.com (K.B.); probalbanerjee@yahoo.com (P.B.); 2 Department of Chemistry, Yunnan Normal University, Kunming 650092, China

**Keywords:** phenyl boronic acid, microgels, tunable onset of glucose responsiveness, volume phase transition, gated insulin retention and release

## Abstract

Glucose-responsive insulin delivery systems that effectively regulate insulin retention and release in response to real-time fluctuation of glucose levels are highly desirable for diabetes care with minimized risk of hypoglycemia. Herein, we report a class of glucose-sensitive copolymer microgels, prepared from a simple one-pot precipitation copolymerization of 4-vinylphenylboronic acid (VPBA), 2-(dimethylamino) ethyl acrylate (DMAEA), and oligo(ethylene glycol) methyl ether methacrylate (M_w_ = 300, MEO_5_MA), for gated glucose-responsive insulin release within the physiologically desirable glucose level range. The composition of the p(VPBA-DMAEA-MEO_5_MA) copolymer microgels were analyzed using NMR and FTIR spectra. The *cis*-diols of glucose can reversibly bind with the −B(OH)_2_ groups of the VPBA component in the microgels, resulting in the formation of negatively charged boronate esters that induce the volume phase transition of the microgels. The DMAEA component is incorporated to reduce the pK_a_ of VPBA, thus improving the glucose sensitivity of the microgels at physiological pH. The neutral hydrophilic MEO_5_MA component is used to tune the onset of the glucose responsiveness of the microgels to the physiologically desirable levels. The more the MEO_5_MA component copolymerized in the microgels, the greater the glucose concentration required to initiate the swelling of the microgels to trigger the release of insulin. When the onset of the glucose response was tuned to 4−5 mM, the copolymer microgels retained insulin effectively in the hypo-/normo-glycemic range but also released insulin efficiently in response to the elevation of glucose levels in the hyperglycemic range, which is essential for diabetes management. The copolymer microgels display no cytotoxicity in vitro.

## 1. Introduction

Diabetes mellitus affects 537 million people worldwide and is projected to rise to 643 million by 2030 [1]. It is a chronic metabolic disorder marked by elevated blood glucose levels (BGL) due to either insulin deficiency from pancreatic β-cell destruction (Type 1) or insulin resistance and progressive β-cell failure (Type 2). Standard treatment for Type 1 and advanced Type 2 diabetes involves frequent BGL monitoring followed by self-injection of insulin [2,3], which poses challenges in terms of compliance and risks of dosing errors. Insulin overdose may cause hypoglycemia, leading to coma or potentially death. To avoid the risk of hypoglycemia, insulin is often underdosed, which can lead to prolonged hyperglycemia and severe complications such as heart disease, kidney failure, and blindness [1,2,3]. To overcome these limitations, there is growing interest in developing glucose-responsive insulin delivery systems that can automatically release insulin in response to hyperglycemia while minimizing premature release under hypoglycemic and normoglycemic conditions.

Polymer microgels have emerged as promising drug carriers due to their biocompatibility, high drug loading capacity, injectable size, and fast response to environmental stimuli [4,5,6,7,8,9]. Various glucose sensing elements have been integrated into polymer microgels, including glucose oxidase (GOx) [10,11,12,13,14], glucose-binding protein concanavalin A (Con A) [15,16,17], and phenylboronic acids (PBA) [18,19,20,21,22,23,24]. While protein-based systems (GOx and Con A) offer biological specificity, they suffer from poor stability, difficult sterilization, and high cost. In contrast, synthetic PBA-based systems offer greater stability and chemical versatility [25], making them attractive for constructing glucose-responsive insulin delivery systems [26,27,28,29,30,31]. The PBA moiety can reversibly bind with the *cis*-diols in glucose to form anionic boronate ester. At pH near the pK_a_ of PBA, glucose binding shifts the equilibrium toward the thermodynamically more favorable anionic form (Figure S1) [18,27], which triggers the microgel swelling to release the encapsulated insulin. Despite this promise, PBA-based delivery systems face several translational challenges, including the toxicity of PBA moieties, response kinetics, glucose specificity, reproducibility, and premature insulin release. Rapid and reversible volume phase transitions are crucial for glucose-responsive insulin delivery, which can be achieved using small-sized PBA-containing microgels [32,33,34,35,36,37,38,39,40,41]. However, other *cis*-diol-containing molecules and ionic species in plasma may interfere with glucose binding. Ionic strength can shield the negative charges of PBA–glucose complexes, reducing responsiveness. We previously evaluated the effects of major plasma constituents, including human serum albumin (44 g/L), L-lactate (20 mM), minor monosaccharides (0.1 mM), and common metal ions (1 mM), and observed a minor perturbation in glucose sensing (within ±7.8% error) [41,42]. Glucose imprinting on the PBA binding sites in microgels can further enhance the glucose specificity and reduce the interference from non-glucose plasma components [42,43]. Still, the biosafety of PBA moieties requires further evaluation [31,44], and scalable consistent synthesis methods for the PBA-based polymer insulin carriers remain a need. Another major unresolved issue is the premature insulin release under hypo-/normoglycemic conditions, as many PBA-based microgels begin to swell at low glucose concentrations [32,33,34,35,36,37,38], increasing the risk of hypoglycemia. To address this challenge, Matsumoto et al. developed a thermo-responsive PBA-containing hydrogel with a metastable dehydrated surface “skin” that releases insulin above a glucose threshold (5.6 mM) [29,45]. While effective, such a bulky gel is unsuitable for real-time injectable insulin releasing systems, due to its slow glucose responsiveness and limited deliverability.

The aim of this work is to develop a class of injectable biocompatible PBA-based copolymer microgels with tunable glucose-responsive thresholds for potential use in a self-regulated insulin delivery system at low risk of hypoglycemia using a simple and benign synthetic method. Unlike previous PBA-based glucose-responsive microgels that often trigger swelling and insulin release at low glucose levels (e.g., below 2 mM) [32,33,34,35,36,37,38], our microgels are designed to shift the glucose responsiveness onset to the physiologically desirable glucose levels. Such threshold gating can minimize insulin release under hypo- and normoglycemic conditions but trigger release under hyperglycemic episodes, thereby representing a critical step toward safe self-regulated insulin therapy. To achieve this, a series of PBA-dominant microgels were prepared using a facile one-pot precipitation copolymerization of 4-vinylphenylboronic acid (VPBA), 2-(dimethyl amino) ethyl acrylate (DMAEA), and oligo(ethylene glycol) methyl ether methacrylate (M_w_ = 300, MEO_5_MA) in water [p(VPBA-DMAEA-MEO_5_MA)]. The major component of VPBA in microgel is designed to provide reversible glucose-responsive functionality. The small amount of DMAEA is used to enhance the glucose sensitivity of the microgels at physiological pH through the B^δ−^…N^δ+^ interactions. The variable amount of MEO_5_MA component is designed to impart hydrophilicity and modulate the glucose thresholds for swelling and insulin release. It has been proven that the −B(OH)_2_ groups in the ortho-methoxyalkyl-substituted PBA molecules form strong hydrogen bonds with nearby ether oxygens (−O−), significantly reducing their ability to bind the *cis*-diols from glucose and other saccharides [46]. In our microgels, the ether oxygens on the oligo(ethylene glycol) (OEG) side chains of the MEO_5_MA component are expected to interact with the −B(OH)_2_ groups of adjacent PBA moieties, hindering glucose access to the –B(OH)_2_ binding sites. To bind these PBA groups and induce microgel swelling, glucose must first disrupt the OEG–PBA interactions via competitive glucose–OEG interactions. As the OEG content increases, more glucose is required to displace the OEG and free the PBA binding sites. Thus, the MEO_5_MA content effectively controls the glucose concentrations needed to initiate glucose–PBA binding, which triggers microgel swelling and insulin release. This design enables selective insulin release only above the desired glycemic thresholds, a key feature for minimizing hypoglycemia risk in glucose-responsive insulin delivery systems.

## 2. Results and Discussion

### 2.1. Synthesis and Compositions of p(VPBA-DMAEA-MEO_5_MA) Microgels

**Figure 1:**
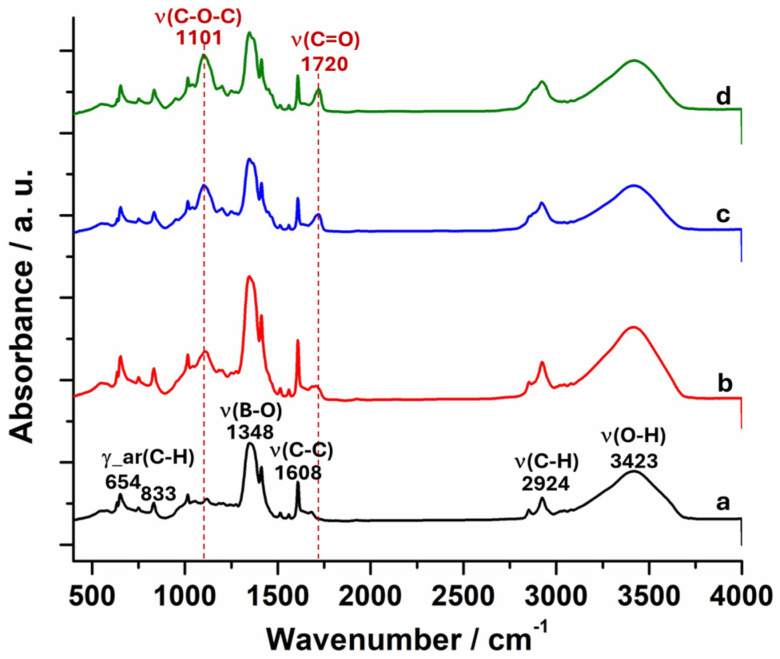
FTIR spectra of the p(VPBA-DMAEA-MEO_5_MA) microgels: (**a**) PBAEO-0, (**b**) PBAEO-1, (**c**) PBAEO-3, and (**d**) PBAEO-4.

The glucose-responsive p(VPBA-DMAEA-MEO_5_MA) microgels were synthesized from a simple one-pot free radical precipitation copolymerization of commercially available VPBA, DMAEA, and MEO_5_MA monomers at different feeding ratios (Table 1), using *N*,*N′*-methylene bisacrylamide (BIS) as a crosslinker. The microgels made with feeding molar ratios of MEO_5_MA/VPBA = 0, 1/10, 2/10, 3/10, and 4/10 were coded as PBAEO-0, PBAEO-1, PBAEO-2, PBAEO-3, and PBAEO-4, respectively. To confirm the successful copolymerization of the monomers, the FTIR spectra of the resultant microgels were collected (Figure 1). The characteristic absorptions of the B−O stretching at 1348 cm^−1^, the phenyl ring C−C stretching at 1608 cm^−1^, and the phenyl ring C−H bending at 833 cm^−1^ indicated the presence of PBA groups in the microgels. The absorption in the range of 1016–1118 cm^−1^ is related to the stretching of the B−C and B−O of PBA groups and the C−N of DMAEA. The characteristic absorptions of the C−H stretching at 2924 cm^−1^ can be attributed to the methylene/methyl groups in the polymer backbone and DMAEA component. In addition to these IR signals from the VPBA and DMAEA components that appeared in the PBAEO-0 sample, the addition of the MEO_5_MA component in the synthesis of PBAEO-1, PBAEO-3, and PBAEO-4 microgels introduced two changes to the IR spectra. One is the characteristic absorption of the C−O−C stretching located at 1101 cm^−1^, and the other is the C=O stretch in ester located at 1720 cm^−1^. Both signals increase gradually with the increase in the feeding ratio of MEO_5_MA/VPBA, indicating the successful incorporation of MEO_5_MA into the microgels.

**Figure 2:**
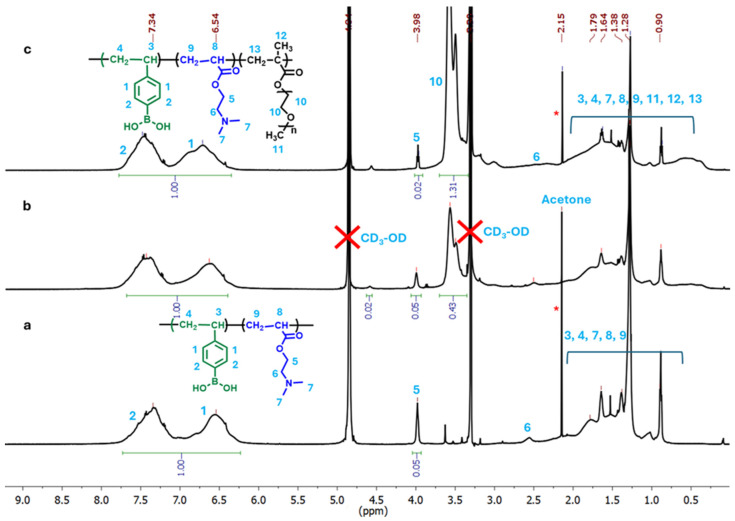
^1^H NMR spectra of the p(VPBA-DMAEA-MEO_5_MA) microgels: (**a**) PBAEO-0, (**b**) PBAEO-1, and (**c**) PBAEO-3 in CD_3_OD. The peak at 2.15 ppm labeled with * is from the residual acetone used to clean the NMR tube.

To determine the compositions of the resulting microgels, ^1^H NMR spectra of the p(VPBA-DMAEA-MEO_5_MA) microgels were measured in CD_3_OD (Figure 2). The chemical shift of the −*CH*_2_− adjacent to the ester group of DMAEA (designated as **5**) at δ = 3.98 ppm and the chemical shifts of the aromatic protons of PBA (designated as **1** and **2**) at δ = 6.54–7.34 ppm were used to determine the ratio of DMAEA/VPBA in the microgel. The observed chemical shift δ = 3.98 ppm for proton **5** of the DMAEA units is slightly upfield compared to the typical δ ≈ 4.15–4.30 ppm for this proton. This deviation likely results from the copolymer microenvironment, restricted chain mobility, and hydrophobic interactions with the nearby aromatic ring of PBA groups within the crosslinked microgel structure. When the −*CH*_2_− protons are located above or below the aromatic ring, they experience a shielding effect. The observed chemical shift δ = 6.54 and 7.34 ppm for protons **1** and **2** in the PBAEO-0 microgels is lower than the typical δ = 7.3–7.9 ppm for the aromatic protons of free VPBA in CD_3_OD. This upfield shift can be attributed to changes in the electronic environment induced by the restricted mobility and altered conjugation of aromatic ring in the crosslinked microgel matrix, as well as the electron-donating effects from the neighboring DMAEA units. The formation of the B…N coordination bond can change the boron hybridization and increase the electron density around the boron atom and subsequently in the aromatic ring. This increased electron density causes the aromatic protons to be more shielded. Through the comparison between the integral area of the proton **5** and the integral area of the aromatic protons (**1** and **2**), the ratio of DMAEA/VPBA in the PBAEO-0 microgel is calculated to be 1/10. This implies that only half of the feeding amount of DMAEA (DMAEA/VPBA feeding ratio = 2/10, Table 1) monomers are copolymerized into microgel. This result is understandable due to the difference in reactivity between the two monomers. In addition, part of the DMAEA monomers might bear a positive charge due to the protonation in the reaction medium (deionized water). These charged DMAEA are hydrophilic, which means they could not be effectively incorporated into the hydrophobic continuously growing p(VPBA-DMAEA) microgel stabilized by the surfactant during the precipitation polymerization reaction. After copolymerizing the MEO_5_MA component into the microgels, new peaks at δ = 3.48 ppm and δ = 3.58 ppm appear in the ^1^H NMR spectra of the PBAEO-1 and PBAEO-3 microgels, which can be attributed to the methylene protons (labelled as **10**) in the ethylene oxide units of MEO_5_MA. The integral areas of the methylene protons **10** in the MEO_5_MA units and the aromatic protons **1** and **2** in the VPBA units enable us to estimate the MEO_5_MA/VPBA ratio copolymerized in the microgel. The determined MEO_5_MA/VPBA ratios of 0.86/10 and 2.6/10 are both smaller than the feeding ratio of 1/10 for PBAEO-1 microgel and 3/10 for PBAEO-3 microgel, respectively, which could be due to the reactivity difference of the two monomers.

### 2.2. Size and Morphology of p(VPBA-DMAEA-MEO_5_MA) Microgels

The hydrodynamic radius (R_h_) of the resultant microgels dispersed in 5 mM PBS of pH = 7.4 was measured using dynamic light scattering (DLS) at 37 °C. The control microgel PBAEO-0 synthesized with only VPBA and DMAEA comonomers displays the smallest size, with the average R_h_ (<R_h_>) = 83 nm. The <R_h_> values of 106 nm, 118 nm, 142 nm, and 148 nm were, respectively, obtained for the microgel samples of PBAEO-1, PBAEO-2, PBAEO-3, and PBAEO-4, indicating that the increase in the feeding amount of the third comonomer MEO_5_MA significantly increased the size of the microgels. The size increase of the microgels at higher feedings of MEO_5_MA should be attributed to the decrease in the feeding ratios of the surfactant sodium dodecyl sulfate (SDS) to the total comonomers. While the feeding amount of MEO_5_MA comonomer was gradually increased from PBAEO-1 to PBAEO-4, the feeding amount of VPBA, DMAEA, and SDS was fixed. In such a situation, the gradually increased total amount of comonomers share the same amount of dispersing agent SDS. Relatively fewer SDS molecules are available for the comonomers, resulting in fewer nuclei particles being formed in the precipitation polymerization process and thus a larger microgel particle size.

**Figure 3:**
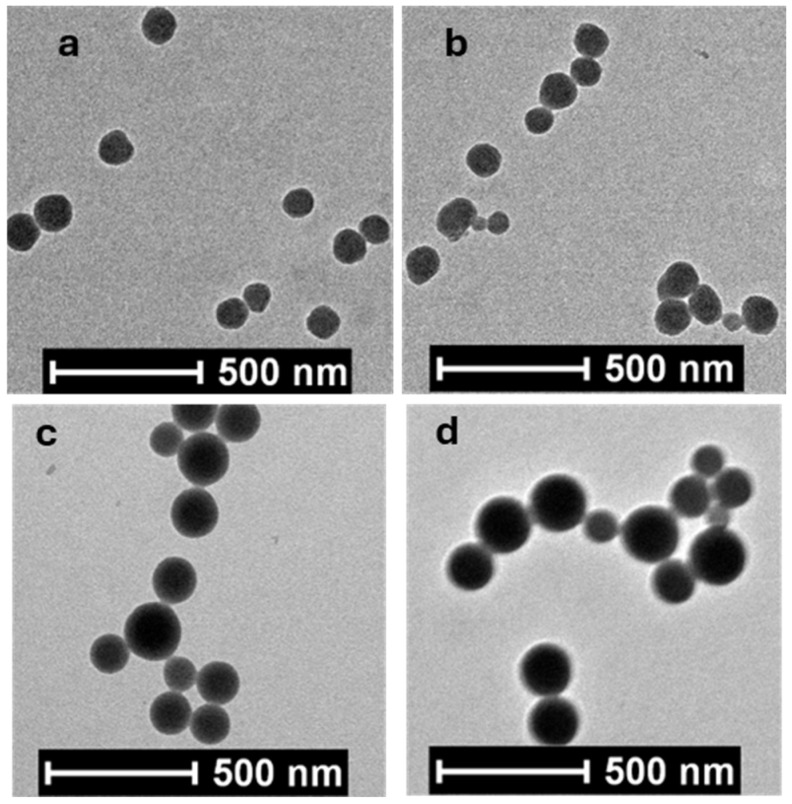
Typical TEM images of the p(VPBA-DMAEA-MEO_5_MA) microgels: (**a**) PBAEO-1, (**b**) PBAEO-2, (**c**) PBAEO-3, and (**d**) PBAEO-4.

Figure 3 shows the typical TEM images of the dried p(VPBA-DMAEA-MEO_5_MA) microgels prepared with different feeding ratios of MEO_5_MA/VPBA. All the microgel particles display a spherical shape. The microgels in a dried state display a much smaller size than their hydrodynamic size. However, the trend of increasing size from PBAEO-1 to PBAEO-4 observed in TEM is consistent with the changes in the <R_h_> measured by DLS. The modest increase in <R_h_> from 106 nm of PBAEO-1 to 118 nm of PBAEO-2 is not readily distinguishable in the dried microgels (Figure 3a,b). Similarly, the slight increase in <R_h_> from 142 nm of PBAEO-3 to 148 nm of PBAEO-4 cannot be easily differentiated either in the TEM images (Figure 3c,d). Nevertheless, the results demonstrate that the size of microgels can be conveniently tuned by varying the feeding ratio of SDS/(total monomers) in the precipitation polymerization method.

### 2.3. pH-Responsive Volume Phase Transition of Microgels

**Figure 4:**
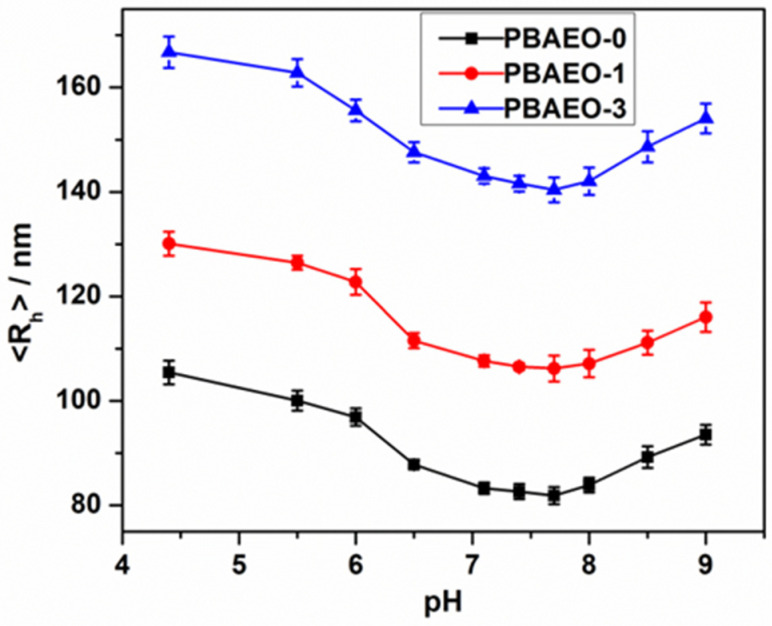
pH-dependent average hydrodynamic radius (<R_h_>) of the microgels PBAEO-0 (■), PBAEO-1 (●), and PBAEO-3 (▲) synthesized with feeding molar ratios of MEO_5_MA/VPBA = 0, 1/10, and 3/10, respectively, dispersed in 5 mM PBS solution. All measurements were made at 37 °C and a scattering angle *θ* = 45°. Error bars indicate mean ± standard deviation (number of replicates n = 3).

Figure 4 shows the pH-induced <R_h_> change in the p(VPBA-DMAEA-MEO_5_MA) microgels, measured at 37 °C. As expected, the size of all three microgels studied increases along both acidic and basic directions. In the acidic pH range, the <R_h_> increases as the pH decreases, because the dimethylamino groups of the DMAEA component will be protonated at pH < pK_b_ (~7.5) of DMAEA, forming positive charges that swell the gel network. In the basic pH range, the PBA moieties will gradually dissociate at pH > pK_a_ of VPBA, forming negative charges that swell the microgels. These results further confirm the successful incorporation of both VPBA and DMAEA components in microgels. The microgels display the smallest size in the pH range of 7.4–7.8, because both the DMAEA and PBA groups exhibited a minimum degree of ionization in this pH range. When pH > 7.8, the size of microgels increases with the increase in pH values due to the dissociation of PBA groups in the microgels. This result indicates that the pK_a_ of PBA groups in the p(VPBA-DMAEA-MEO_5_MA) microgels has been lowered to 7.8 compared to the pK_a_ ~ 8.8 of free VPBA [47], which is important to enhance the glucose sensitivity of the microgels at the physiological pH. The reduced pK_a_ of the PBA groups in these p(VPBA-DMAEA-MEO_5_MA) microgels are realized via the B^δ−^…N^δ+^ interactions between the neighboring PBA groups and DMAEA groups.

### 2.4. Tuning the Glucose-Responsive Volume Phase Transitions

**Figure 5:**
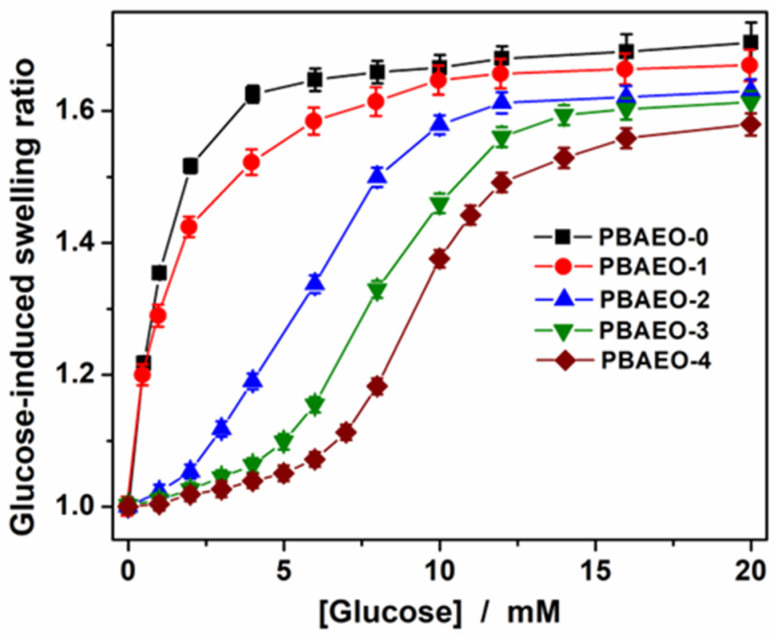
Glucose-induced swelling ratios of the p(VPBA-DMAEA-MEO_5_MA) copolymer microgels based on the <R_h_>_[Glu]_/<R_h_>_0.0 mM_, with <R_h_>_0.0 mM_ and <R_h_>_[Glu]_ being the average hydrodynamic radius <R_h_> measured in PBS of pH 7.4 at 0.0 mM glucose and a series of different glucose concentrations, respectively. PBAEO-0, PBAEO-1, PBAEO-2, PBAEO-3, and PBAEO-4 represent the microgels synthesized with feeding molar ratios of MEO_5_MA/VPBA = 0, 1/10, 2/10, 3/10, and 4/10, respectively. All measurements were made at 37 °C and a scattering angle θ = 45°. Error bars indicate mean ± standard deviation propagated from errors of <R_h_> measurements (number of replicates n = 3).

PBA-containing microgels show reversible glucose-responsive size change because the *cis*-diols of glucose can bind with the PBA groups in the gel network chains, forming negatively charged complexes [18,28]. Our goal was to tune this response to the physiologically desirable glucose range by copolymerizing varying amounts of MEO_5_MA into the PBA-dominant microgels. Figure 5 shows the glucose-induced swelling curves of the p(VPBA-DMAEA-MEO_5_MA) microgels, in terms of the ratio <R_h_>_[Glu]_/<R_h_>_0.0 mM_, with <R_h_>_[Glu]_ and <R_h_>_0.0 mM_ being the <R_h_> values measured in PBS (pH 7.4, 37 °C) at different glucose concentrations and no glucose, respectively. First, the increase in the content of MEO_5_MA component gradually reduces the maximum swelling degree. This is expected, because the higher content of MEO_5_MA in the microgels decreases the relative amount of the glucose-sensitive PBA units, thus lowering the extent of ionization of network chains and swelling degree. Nevertheless, the glucose-induced maximum swelling ratios of all the copolymer microgels are above 1.5 even for the PBAEO-4 sample with the lowest VPBA content, which is sufficient to trigger insulin release. Second, the increase in the MEO_5_MA content shifts the onset of swelling toward higher glucose concentrations. For example, both PBAEO-0 prepared without MEO_5_MA and PBAEO-1 synthesized with a low feeding ratio of MEO_5_MA/VPBA = 1/10 exhibited a sharp increase in <R_h_> in response to the addition of glucose even at concentrations below 1 mM. In contrast, the PBAEO-2, PBAEO-3, and PBAEO-4 microgels, synthesized with the feeding ratios of MEO_5_MA/VPBA = 2/10, 3/10, and 4/10, shifted the onset of glucose-responsive volume phase transitions to the glucose concentrations of 2.0 mM, 4.0 mM, and 5.0 mM, respectively. This tunable onset of glucose-responsive volume phase transition at the physiologically desirable glucose levels is critical to develop PBA-based microgels for self-regulated insulin delivery, as it can minimize insulin release under hypo- and normoglycemic conditions. Our results demonstrate that this is achievable by rationally designing the PBA-based microgels with different MEO_5_MA contents.

The MEO_5_MA polymer segments in the microgels remain neutral and hydrophilic at the physiological temperature of 37 °C (well below its LCST) [48,49,50]. In the absence of glucose, the ether oxygens on the flexible OEG side chains of the MEO_5_MA component can form hydrogen bonds with the neighboring PBA groups in the microgels (–B(OH)_2_…OEG) [46]. This OEG–PBA interaction is supported by the systematic downfield shift of phenyl protons **1** and **2** in the NMR spectra of PBAEO-0, PBAEO-1, and PBAEO-3 microgels (Figure 2). When glucose is added into these MEO_5_MA-containing microgel solutions, glucose molecules must first disrupt the OEG–PBA interactions through competitive glucose–OEG hydrogen bonding interactions to access the PBA binding sites. Strong and extensive hydrogen bonding interactions between the ether oxygens (−O−) in OEG or polyethylene glycol (PEG) and the hydroxyl (−OH) groups in glucose have been experimentally evidenced [51,52,53]. These glucose–PEG interactions can modulate the PEG–water interactions and change the phase behavior of the PEG solution [52,53,54]. In our microgels, the disruption of OEG–PBA adducts via the glucose–OEG interactions does not trigger microgel swelling. The hypothesis that the OEG–PBA interactions hinder glucose access to the PBA binding sites in our microgels can be supported by prior studies showing that strong hydrogen bonding between the ether oxygen and boronic acid in the ortho-methoxyalkyl-substituted PBA molecules dramatically reduces the glucose binding affinity [46]. As the MEO_5_MA content increases, the extent of OEG-PBA interactions inside the microgels also rises, requiring more glucose to displace OEG and free the PBA groups. Only after this displacement, glucose can bind to the freed PBA and form anionic boronate esters, triggering microgel swelling. Thus, the MEO_5_MA content in the microgels can modulate the glucose threshold to induce the volume phase transition.

**Figure 6:**
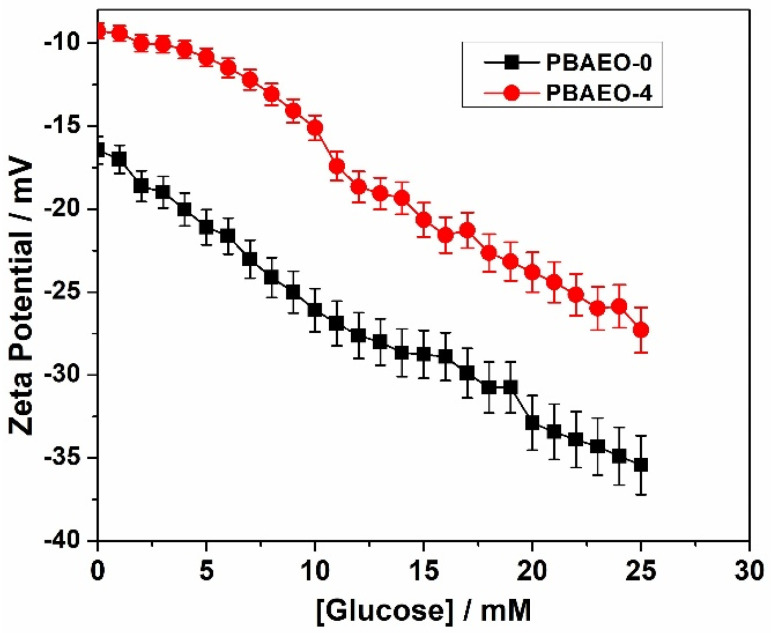
Zeta potential of the p(VPBA-DMAEA-MEO_5_MA) copolymer microgels PBAEO-0 (■) and PBAEO-4 (●) synthesized with feeding molar ratios of MEO_5_MA/VPBA = 0 and 4/10, respectively, dispersed in 5 mM PBS solution of pH = 7.4 with different glucose concentrations. All measurements were made at room temperature of 22 °C. Data are the mean from two repeated measurements. Error bars represent ± 5% instrumental accuracy error.

Figure 6 shows the glucose-dependent Zeta potential of the PBAEO-0 and PBAEO-4 microgels. In the absence of glucose, both microgels carry negative surface charges, with the Zeta potentials ζ = −16.5 mV for PBAEO-0 and ζ = −9.3 mV for PBAEO-4, respectively. These initial negative surface charges of the microgels likely arise from the partial dissociation of PBA groups (due to a lowered pK_a_ in the copolymer microgels) and residual SDS molecules adsorbed on the VPBA-dominant microgel surface after week-long dialysis. In comparison with the PBAEO-0 microgel, the PBAEO-4 microgel exhibits two distinguishable features in the glucose-dependent Zeta potential curve. First, the onset of the glucose-responsive Zeta potential change is different for the two microgels. The PBAEO-0 microgel containing no MEO_5_MA component shows an immediate and progressive change in Zeta potential once the glucose concentration starts to increase, even at the low range, indicating readily accessible PBA groups for glucose molecules to bind, forming anionic glucose–PBA complexes. The higher the glucose concentration, the more negative the Zeta potential, because more anionic glucose–PBA complex is formed. In contrast, the PBAEO-4 microgel shows minimal change in the Zeta potential in the glucose concentration range of 0–5 mM. Only when the glucose concentration is above 5 mM (onset) does the glucose-induced drop in Zeta potential become significant. This result suggests that no significant additional negative charges are generated between 0 and 5 mM of glucose. The high MEO_5_MA content in the PBAEO-4 microgel leads to extensive OEG–PBA adducts, requiring elevated glucose levels (>5 mM) to competitively disrupt these OEG–PBA interactions and free the PBA groups for anionic glucose–PBA complex formation. The glucose-responsive Zeta potential changes observed in PBAEO-0 and PBAEO-4 microgels correlate well with their glucose-responsive swelling behavior (Figure 5), as swelling is driven by the formation of anionic glucose–PBA complexes. Second, the PBAEO-4 microgel exhibits a significantly less negative Zeta potential than the PBAEO-0 microgels at all the glucose concentrations. This is likely due to the incorporation of a substantial amount of neutral hydrophilic MEO_5_MA polymer segments in the PBAEO-4 microgel, diluting the surface charges resulted from the anionic glucose–PBA complexes.

To further confirm the OEG effects on the glucose–PBA binding ability, we collected the UV–Vis absorption spectra for the PBAEO-0 and PBAEO-3 microgels in the presence of 0, 3 mM, and 10 mM glucose, respectively (Figure S2). It has been reported that the formation of anionic boronate esters in PBA-containing polymer will slightly diminish the PBA absorbance intensity [55]. Our results also show the decrease in PBA absorbance as the glucose–PBA boronate complexes form in microgels. However, the relative absorbance change (ΔA) is different for the two microgels. In PBAEO-0, ΔA is significant when glucose changes from 0 to 3 mM, indicating the formation of anionic boronate ester at 3 mM glucose. In contrast, in PBAEO-3, ΔA is very small when glucose changes from 0 to 3 mM, but significant ΔA is observed as glucose rises from 3 mM to 10 mM, implying that the amount of anionic boronate ester is minimal at 0–3 mM glucose but increases significantly as glucose changes from 3 to 10 mM. These results further support that glucose can readily access the PBA binding sites in OEG-free PBAEO-0 microgel, but glucose must disrupt the OEG–PBA interactions in PBAEO-3 microgel to free the PBA to form anionic glucose–PBA complexes.

### 2.5. Insulin Loading and In Vitro Glucose-Regulated Insulin Release

After confirming the tunable onset of glucose-responsive volume phase transitions of the microgels, three microgel samples of PBAEO-1, PBAEO-3, and PBAEO-4 were selected to load the insulin drug. The porous network structure of the microgels, combined with the hydrogen bonding and hydrophobic interactions between the insulin molecules and microgel network chains, makes them well suited for encapsulating insulin, resulting in a high drug loading capacity. The loading capacity (expressed as the mass of loaded insulin per unit weight of dried microgels) of the microgels for FITC-insulin was determined to be 33.3, 35.9, and 37.0 wt% for PBAEO-1, PBAEO-3, and PBAEO-4, respectively, which is much higher than the typical loading capacities (~7–20 wt%) obtained in other PBA-containing nano-/microgel insulin carriers [34,35,36,37,38,39,40], but it is similar to those obtained in our previously developed VPBA-based microgels [32,33]. A high insulin loading capability is desirable for insulin carriers, as it may reduce the frequency of injections if used in vivo.

**Figure 7:**
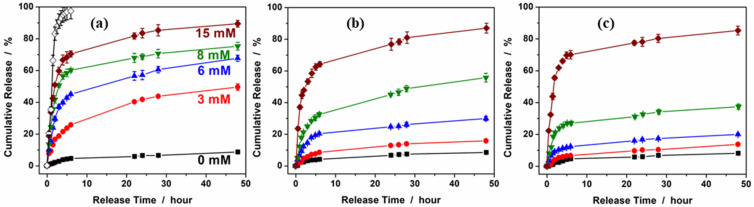
Releasing profiles of FITC-insulin from (**a**) PBAEO-1, (**b**) PBAEO-3, and (**c**) PBAEO-4 microgels synthesized with feeding molar ratios of MEO_5_MA/VPBA = 1/10, 3/10, and 4/10, respectively, in the presence of 0 (■), 3 (●), 6 (▲), 8 (▼), and 15 (♦) mM glucose in PBS of pH 7.4. In the blank release (◊), the release experiment of the FITC-insulin solution with an equivalent amount of drug to that trapped in PBAEO-1 was performed in PBS of pH 7.4. All experiments were carried out at 37 °C. Error bars represent 95% confidence interval.

Figure 7 shows the glucose-responsive insulin release profiles from the PBAEO-1, PBAEO-3, and PBAEO-4 microgels measured using the dialysis method for 48 h in PBS of pH 7.4 at 37 °C, respectively. A blank release experiment using a free FITC-insulin solution containing an equivalent amount of drug to that trapped in PBAEO-1 was also performed. This control experimental result shows that the dialysis membrane had a negligible influence on the releasing kinetics. First, all three microgels display glucose-responsive insulin release because of their glucose-responsive volume phase transitions (or swelling). As the glucose concentration in the releasing medium increases, more insulin molecules are released from the microgels. This is because higher glucose levels lead to the formation of more negatively charged glucose–PBA complexes within the microgels, causing them to swell more. The resultant enlargement of the mesh size of the microgels allows the encapsulated insulin molecules to diffuse out more easily. In the absence of glucose, only 8.8%, 8.5%, and 8.2% of the loaded insulin was released from the PBAEO-1, PBAEO-3, and PBAEO-4 microgels, respectively, after 48 h in PBS (pH 7.4) buffer solution, indicating that these microgels can retain insulin effectively. The small amount of insulin release (8.2–8.8%) from the microgels in the buffer solution is understandable because insulin molecules located in the peripherical region can slowly diffuse out due to the concentration gradient. Second, the onset of the glucose-responsive volume phase transition of the microgels is critical to determine at which glucose levels insulin should be retained and released. The PBAEO-1 microgel starts to swell at very low glucose concentrations (e.g., 0.5 mM) (Figure 5), thus it can release a significant amount of insulin even at low glucose levels. For example, 49.6% and 67.4% of the loaded insulin are released from the PBAEO-1 microgel at 3.0 mM and 6.0 mM of glucose, respectively, after 48 h (Figure 7a). Such high amounts of insulin release in the hypoglycemic (e.g., 3 mM) and normoglycemic (e.g., 6 mM) range could cause severe hypoglycemia if used for diabetic care. In contrast, the PBAEO-3 microgel, with an onset of glucose-responsive volume phase transition tuned to 4.0 mM, releases much less insulin in the hypo- and normoglycemic range. Only 16.0% and 29.8% of the loaded insulin can be released after 48 h at 3.0 mM and 6.0 mM of glucose, respectively (Figure 7b). Evidently, the insulin release at low and normal glucose levels is dramatically reduced, which is desirable for insulin carriers. Further tuning the onset of glucose responsiveness to 5.0 mM in the PBAEO-4 microgels can control the retention and release of insulin even more effectively. Only 13.9% and 19.9% (or 5.7% and 11.7% after correction of the baseline at 0 mM glucose) of the loaded insulin can be released after 48 h from the PBAEO-4 microgels at 3.0 mM and 6.0 mM of glucose (Figure 7c), respectively, demonstrating excellent insulin retention ability of the microgels in the hypo- and normo-glycemic ranges. On the other hand, when glucose levels rise into the hyperglycemic range, the PBAEO-4 microgels respond effectively by releasing an appropriate amount of insulin. For example, 37.5% of the loaded insulin is released after 48 h at 8.0 mM of glucose, and this further increases to 85.2% under severe hyperglycemia (15 mM glucose). Our results from the PBAEO-3 and PBAEO-4 microgels with onset of glucose responsiveness at ~4 mM and ~5 mM, respectively, show that tuning the onset of glucose-sensitive volume phase transition of microgels to the desired glucose threshold is essential to achieve optimal insulin retention and release. The minimal insulin release from these microgels at the hypoglycemic and normoglycemic levels (e.g., 3 and 6 mM) can significantly reduce the risk of hypoglycemia. Yet, the glucose-regulated fast insulin release from the microgels at high glucose levels will treat hyperglycemia effectively. It is important to balance the safety (hypoglycemia avoidance) and efficacy (timely insulin release in hyperglycemic range). An excessively high glucose threshold could potentially delay the therapeutic response during the early stages of hyperglycemia. In our microgels, we can tune the onset of the glucose-responsive swelling and insulin release from ~1 mM (PBAEO-2) up to ~5 mM (PBAEO-4) by varying the MEO_5_MA content. While PBAEO-4 (~5 mM onset) shows excellent insulin retention and avoids premature insulin release under normoglycemia, PBAEO-3 (~4 mM onset) may be more suitable when early therapeutic response is prioritized. This design flexibility allows for the selection of formulations based on treatment goals to balance safety and efficacy. The sustained release over 48 h may also meet the basal insulin needs. Basal insulin requires a slow constant insulin release to mimic the continuous low-dose insulin supply of a healthy pancreas. Medically available long-acting insulin formulations specifically designed for basal needs can last for about 20–36 h with Glargine and up to 42 h with Tresiba. Based on the insulin-releasing profiles of our microgels, continuous steady release beyond 48 h in the normoglycemia range is expected for sample PBAEO-3. It is possible to extend the release profiles for ultralong (e.g., 96 h) basal insulin supply with further fine tuning on the crosslinking density and glucose responsiveness onset of the microgels due to its structure stability. Furthermore, sample PBAEO-3 with glucose responsiveness onset ~ 4 mM can be a promising candidate to combine both basal and bolus needs, because it shows a slow steady insulin release in the normoglycemia range (~4–5.5 mM) and a fast release under hyperglycemic conditions.

A fast and reversible insulin release rate in response to the glucose level fluctuation is critical for the glucose-responsive insulin delivery. Here, we selected the PBAEO-3 microgel with glucose onset ~4 mM as a platform to test the reversibility of “On–Off” insulin release by exposing the microgels alternately to 3 mM (hypoglycemia) and 15 mM (hyperglycemia) glucose for 50 min at each glucose level for three cycles. As shown in Figure S3, when the insulin-loaded microgels were exposed to PBS solution containing 3 mM glucose, the insulin release rate was very slow (shut off). Once the microgels were exposed to the PBS solution containing 15 mM glucose, the insulin release rate returned to fast quickly (switch on). These results obtained from the insulin-loaded PBAEO-3 microgel demonstrate the glucose-triggered On–Off insulin release. These insulin release characteristics from the newly designed p(VPBA-DMAEA-MEO_5_MA) microgels confer advantages for self-regulated insulin delivery systems.

### 2.6. In Vitro Cytotoxicity

**Figure 8:**
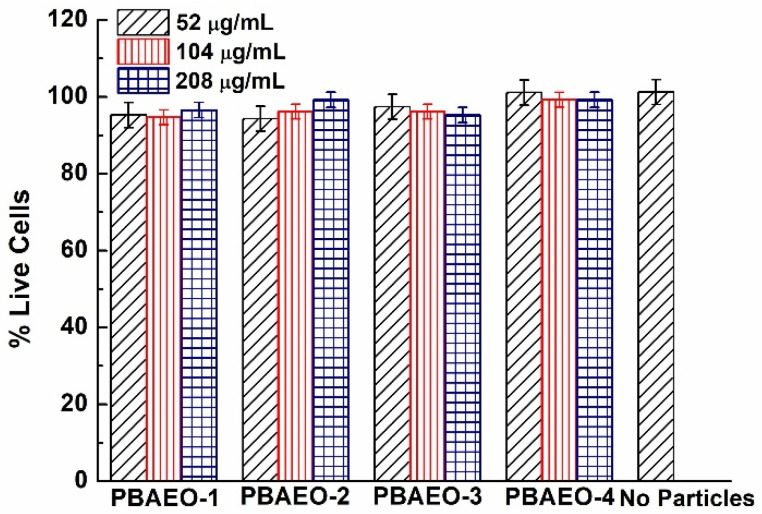
Cell viability of the p(VPBA-DMAEA-MEO_5_MA) copolymer microgels containing different amounts of the MEO_5_MA component.

The cytotoxicity of the p(VPBA-DMAEA-MEO_5_MA) copolymer microgels was tested by quantifying the cellular metabolic activity using an MTT assay. Figure 8 shows the cellular metabolic activity of B16F10 cells treated with the PBAEO-1, PBAEO-2, PBAEO-3, and PBAEO-4 microgels at different concentrations in the culture medium, respectively. As expected, the p(VPBA-DMAEA-MEO_5_MA) microgels showed little toxicity to the B16F10 cells after incubation for 24 h at concentrations up to 208 μg/mL. The PBAEO-4 microgel sample containing more MEO_5_MA polymer segments seemed to further improve the cellular metabolic activity due to the biocompatibility of the OEG component. Although the current study only evaluated the cytotoxicity of the p(VPBA-DMAEA-MEO_5_MA) microgels on B16F10 cells for 24 h, it should be noted that our previous cytotoxicity study of structurally similar and size comparable poly(acrylamide-co-VPBA) microgels on three cell lines over a 72 h period demonstrated minimal cytotoxicity across all the tested cell lines (B16F10, 4T1, and HEK293T) [33]. We expect the current microgels to exhibit similarly low cytotoxicity.

## 3. Materials and Methods

### 3.1. Materials

D(+)-glucose was purchased from ACROS, and all other chemicals were purchased from Aldrich. 2-(dimethyl amino) ethyl acrylate (DMAEA) and oligo(ethylene glycol) methyl ether methacrylate (M_n_ = 300 g/mol, MEO_5_MA) were purified with neutral Al_2_O_3._ The lyophilized fluorescein isothiocyanate-labeled insulin (FITC-insulin) from bovine pancreas (~5800 Da), 4-vinylphenylboronic acid (VPBA), *N*,*N′*-methylene bisacrylamide (BIS), 2,2′-azobis(2-methylpropionamidine) dihydrochloride (AAPH), sodium dodecyl sulfate (SDS), Dulbecco’s modified eagle medium (DMEM), and fetal bovine serum (FBS) were used as received without further purification.

### 3.2. Synthesis of p(VPBA-DMAEA-MEO_5_MA) Microgels

The detailed procedure for synthesis of microgels was described in our previous work [56]. In a 250 mL round-bottom flask equipped with a stirrer, a N_2_ gas inlet, and a condenser, 1.63 mmol of VPBA was added to 100 mL deionized water and heated to 70 °C. After the VPBA was completely dissolved, 0.329 mmol of DMAEA, 0.002 g of BIS, 0.351 mmol of SDS, and the desired amount of MEO_5_MA were successively added to the solution under stirring. Following a continuous N_2_ purge at 70 °C for 30 min, the copolymerization was initiated by adding 1 mL of 0.105 M AAPH. The polymerization reaction was allowed to proceed for 5 h. The reaction product was centrifuged at 6000 rpm for 30 min (using a Thermo Electron Co. SORVALL^®^ RC-6 PLUS super-speed centrifuge, Waltham, MA, USA), the supernatant was discarded, and the precipitate was redispersed in 100 mL of deionized water. The resultant copolymer microgels were further purified by dialysis for seven days against very frequently changed water at room temperature (~22 °C).

### 3.3. Dynamic Light Scattering (DLS) Studies

DLS was performed on a BI-200SM light scattering spectrometer equipped with a BI-9000 AT digital time correlator (Brookhaven Instruments Corp., Holtsville, NY, USA). Am Nd:YAG laser (150 mW, 532 nm) was used as the light source. All microgel solutions were filtered through a Millipore Millex-HV filter (0.80 µm) to remove dust before the measurements. In DLS, the Laplace inversion of each measured intensity–intensity time correlated function can result in a characteristic line width distribution *G*(*Γ*). For a purely diffusive relaxation, *Γ* is related to the translational diffusion coefficient D by (*Γ*/*q^2^*)*_C_*_→0,*q*→0_ = D, where *q* = (4*πn/λ*)sin(*θ*/2) with *n*, *λ*, and *θ* being the solvent refractive index, the wavelength of the incident light in vacuo, and the scattering angle, respectively. *G*(D) can be further converted to a hydrodynamic radius (R_h_) distribution by using the Stokes–Einstein equation, R_h_ = (*k*_B_*T/6πη*)*D*^−1^, where *k*_B_, *T*, and *η* are the Boltzmann constant, the absolute temperature, and the solvent viscosity, respectively [57].

### 3.4. Insulin Loading and In Vitro Release

FITC-insulin was loaded into microgels using a complexation method [56]. A stock solution of FITC-insulin (1 mg/mL) was prepared in 5 mM PBS of pH 7.4 and stored in a refrigerator at 4 °C. The pH of the microgel dispersion (5 mL) was adjusted to 9.0 using a dilute NaOH solution. This dispersion was stirred in an ice water bath for 30 min; then, 1 mL of FITC-insulin solution was added dropwise to the vial. The immediate clouding phenomenon indicates the complexation interaction of the insulin molecules with the microgel network chains. After stirring overnight, the suspension was centrifuged at 6000 rpm for 30 min. To remove free drug molecules, the precipitate was redispersed in 5 mL of PBS at pH 7.4 and further purified by repeated centrifugation and washing. Finally, the precipitate of insulin-loaded microgels was redispersed in 1 mL of PBS of pH 7.4. All the upper clear solutions were collected. The concentration of free FITC-insulin was determined by fluorescence spectrometry at 518 nm upon excitation of 492 nm. The amount of loaded insulin in the microgels was calculated from the decrease in drug concentration. The loading capacity is expressed as the mass of loaded drug per unit weight of dried microgels. The in vitro release of FITC-insulin from the microgels was evaluated using the dialysis method. A dialysis bag filled with 1 mL of purified insulin-loaded microgel dispersion was immersed in 50 mL of 5 mM PBS of pH 7.4, with varying glucose concentrations. Samples of the released FITC-insulin outside the dialysis bag were taken at defined time intervals and assayed by fluorescence spectrometry. The cumulative release is expressed as the total percentage of insulin released through the dialysis membrane over time.

### 3.5. In Vitro Cytotoxicity Studies

B16F10 cells (2000 cell/well) were cultured in DMEM containing 10% FBS and 1% penicillin–streptomycin in a 96-well plate and exposed to the microgels PBAEO-(1–4), respectively. To cover the high concentrations, the microgels were concentrated and adjusted to an appropriate concentration in DMEM right before being added into the wells. The plate was incubated at 37 °C for 24 h. The medium was then aspirated. These wells were washed using fresh serum-free DMEM, followed by the addition of 25 μL of 3-(4,5-dimethyl-2-thiazolyl)-2,5-diphenyltetrazolium bromide (MTT) solution (5 mg/mL in PBS). After a 2 h incubation, the solution was aspirated, and 100 μL of dimethyl sulfoxide was added to each well. The plate was sealed and incubated for 30 min at 37 °C with gentle mixing. Three portions of the solution obtained from each well were transferred to the three respective wells of a 96-well plate. The cellular metabolic activity was measured using a microplate reader at 570 nm. Positive controls contained no microgels, and negative controls contained MTT.

### 3.6. Other Characterizations

The morphology of the microgels was characterized by transmission electron microscopy (TEM) on an FEI TECNAI transmission electron microscope at an accelerating voltage of 120 kV. Approximately 10 µL of filtered dilute microgel suspension was dropped on a Formvar-covered copper grid and then air-dried at room temperature for the TEM measurements. The FTIR spectra were recorded with a Thermo Scientific Nicolet 6700 Fourier transform infrared spectrometer. The NMR spectra were obtained via a Varian VNMRS 600 MHz Fourier transform nuclear magnetic resonance spectrometer. The purified and lyophilized microgel samples were dissolved in the deuterated methanol (CD_3_OD) for NMR measurements. The PL spectra were respectively obtained on a JOBIN YVON Co. FluoroMax^®^-3 Spectro fluorometer (HORIBA Scientific, Edison, NJ, USA), equipped with a Hamamatsu R928P photomultiplier tube, calibrated photodiode for excitation reference correction from 200 to 980 nm, and an integration time of 1 s. The Zeta potential measurements of the microgels were carried out on Microtrac’s Stabino Zeta (Microtrac Inc., Haan, Germany) using a 400 micrometer Piston. The pH values were obtained on a METTLER TOLEDO SevenEasy pH meter (Columbus, OH, USA).

## 4. Conclusions

A series of p(VPBA-DMAEA-MEO_5_MA) copolymer microgels with tunable onset of glucose-responsive volume phase transitions can be prepared using the facile one-pot precipitation polymerization. The incorporation of about 10 mol% DMAEA in the microgels can lower the pK_a_ of PBA to about 7.8 in the VPBA-dominant copolymer microgels. The OEG side chains of the MEO_5_MA component can interact with the neighboring PBA groups inside the microgels, which makes the PBA groups not easily accessible for binding glucose. The added glucose molecules need to break the OEG–PBA interaction via competitive hydrogen bonding with the OEG chains before binding to the PBA groups. The higher the MEO_5_MA component in the microgels, the higher the glucose concentration required to initiate the glucose–PBA binding that swells the microgels. When the onset of the glucose-responsive volume phase transition is tuned to the desired physiologically important glucose levels (e.g., ~4–5 mM), the PBAEO microgels can retain insulin effectively in the hypo- and normoglycemic ranges but release appropriate amounts of insulin in response to increases in glucose concentration in the hyperglycemic range, which is essential to develop self-regulated insulin delivery systems. The microgel particles demonstrated no cytotoxicity in vitro.

## Figures and Tables

**Table 1:** Feeding amount of monomers for synthesis of p(VPBA-DMAEA-MEO_5_MA) microgels.

Sample	VPBA (mmol)	DMAEA (mmol)	MEO_5_MA (mmol)	Molar Ratio
PBAEO-0	1.63	0.329	−	10/2/0
PBAEO-1	1.63	0.329	0.163	10/2/1
PBAEO-2	1.63	0.329	0.326	10/2/2
PBAEO-3	1.63	0.329	0.489	10/2/3
PBAEO-4	1.63	0.329	0.652	10/2/4

## Data Availability

The original contributions presented in this study are included in the article. Further inquiries can be directed to the corresponding authors.

## Supplementary Materials

The following supporting information can be downloaded at https://www.mdpi.com/article/10.3390/molecules30153059/s1, Figure S1: Complexation equilibrium between boronic acid and glucose in aqueous solution; Figure S2: UV–Vis spectra of PBAEO-0 and PBAEO-3 microgels under different glucose concentrations; Figure S3: On–off release of insulin from PBAEO-3 microgels triggered by 15 mM and 3 mM glucose, alternately.
