# Supplementary material for: One-Pot Synthesis of Phenylboronic Acid-Based Microgels for Tunable Gate of Glucose-Responsive Insulin Release at Physiological pH
Source: Molecules. 2025 Jul 22;30(15):3059. doi: 10.3390/molecules30153059 (PMC12348508; doi:10.3390/molecules30153059)
Supplement: Supplementary file 1 [file molecules-30-03059-s001.zip › molecules-3720838-supplementary.pdf]

Article

One-pot Synthesis of Phenylboronic Acid-Based Microgels for Tunable Gate of Glucose-Responsive Insulin Release at Physiological pH

Prashun G. Roy[1,†], Jiangtao Zhang[1,†], Koushik Bhattacharya[1], Probal Banerjee[1], Jing Shen[1,2,*], and Shuiqin Zhou[1,*]

[1]Department of Chemistry of The College of Staten Island, and The PhD Program in Chemistry of Graduate Center, The City University of New York, 2800 Victory Boulevard, Staten Island, NY 10314
[2]Department of Chemistry, Yunnan Normal University, Kunming 650092, China
* Correspondence: shenjingbox0225@hotmail.com (J. S.); shuiqin.zhou@csi.cuny.edu (S.Z.)
† These authors contributed equally to this work.

Supplementary Materials. Table of Content

Figure S1. Complexation equilibrium between the boronic acid and glucose in aqueous solution.

Figure S2. UV-Vis spectra of the PBAEO-0 and PBAEO-3 microgels under 0 mM, 3 mM, and 10 mM glucose in PBS solution of pH 7.4.

Figure S3. On-off release of insulin from the PBAEO-3 microgels triggered by 15 mM and 3 mM glucose alternatively in PBS solution of pH 7.4.

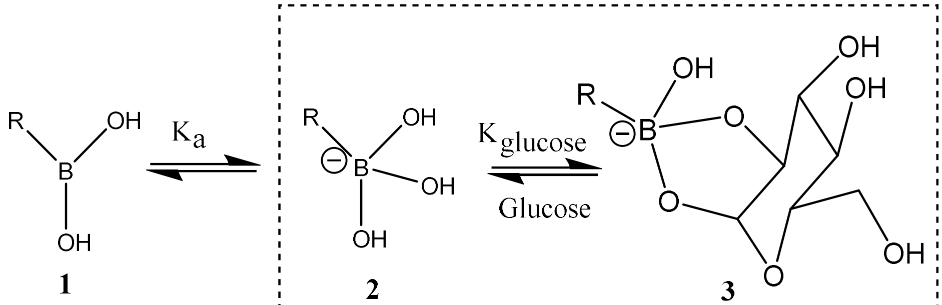

1 **2** **3**

Figure S1. Complexation equilibrium between the boronic acid and glucose in aqueous solution: Boronic acid shows equilibrium with its undissociated neutral trigonal form (1) and a dissociated anionic tetrahedral form (2) in water. The cyclic boronic esters between a *cis*-diol and a neutral boronic acid (1) are hydrolytically unstable. However, when the 1, 2-*cis*-diol or 1,3-*cis*-diol of glucose molecule encounter with the anionic form of boronic acid (2), they reversibly bond together and form a stable 5- or 6-membered ring of cyclic boronate ester (3), thus shifting the equilibrium to the anionic form favorably (3).

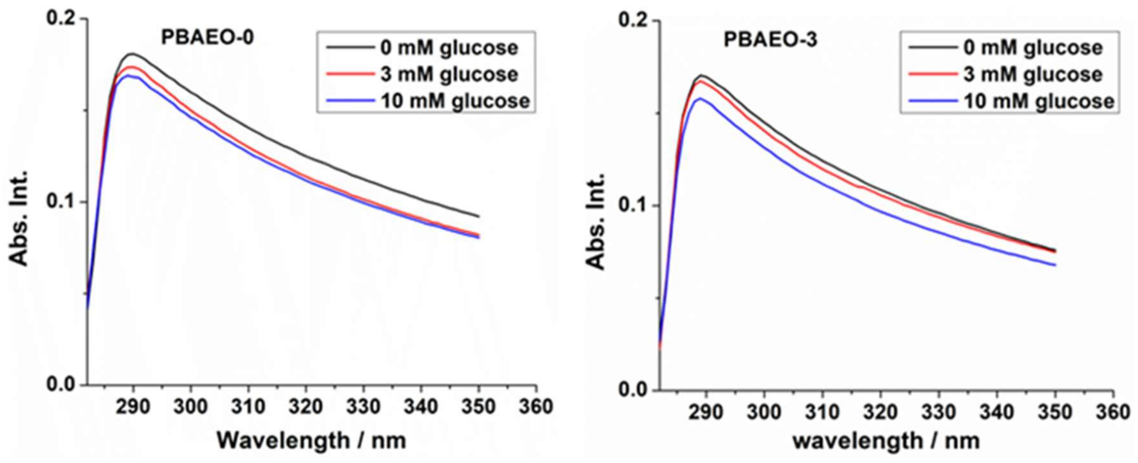

Figure S2. UV-Vis absorption spectra of PBAEO-0 and PBAEO-3 microgels synthesized with feeding molar ratios of MEO_5MA/VPBA = 0 and 3/10, respectively, dispersed in 5 mM PBS solution of pH = 7.4 at different glucose concentrations. The spectra were collected at room temperature.

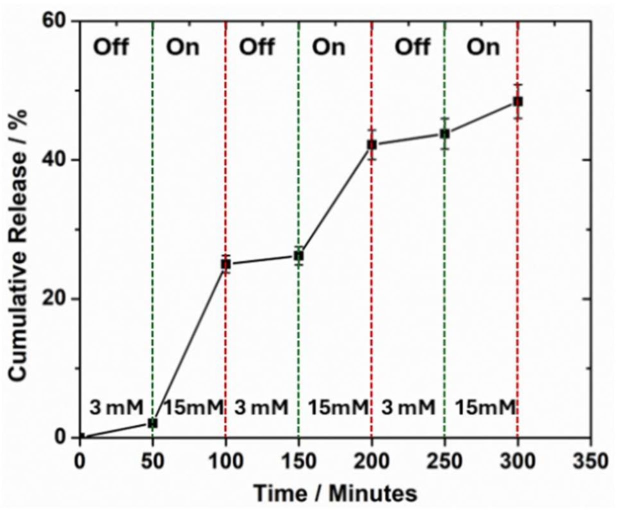

Figure S3. On-off release of insulin from the PBAEO-3 microgels synthesized with feeding molar ratios of MEO_5MA/VPBA = 3/10, obtained by exposing the microgels alternately to 3 mM (hypoglycemia) and 15 mM (hyperglycemia) glucose for 50 minutes at each glucose level for three cycles in 5 mM PBS solution of pH = 7.4. Error bars represent mean ± SD.
